# Scenes From Tick Physiology: Proteins of Sialome Talk About Their Biological Processes

**DOI:** 10.3389/fcimb.2021.767845

**Published:** 2022-01-04

**Authors:** Natalia Fernández-Ruiz, Agustín Estrada-Peña

**Affiliations:** ^1^ Faculty of Veterinary Medicine, University of Zaragoza, Zaragoza, Spain; ^2^ Group of Research on Emerging Zoonoses, Instituto Agroalimentario de Aragón (IA2), Zaragoza, Spain

**Keywords:** tick, proteins, salivary glands, biological process, networks, PageRank

## Abstract

Ticks are blood-sucking parasites with different strategies of feeding depending on the tick family. The major families are Ixodidae or Argasidae, being slow or fast feeders, respectively. In the recent years, the advances in molecular sequencing techniques have enabled to gain knowledge about the proteome of the tick’s salivary glands. But an holistic view of the biological processes underlying the expression of the sialome has been neglected. In this study we propose the use of standard biological processes as a tool to draw the physiology of the tick’s salivary glands. We used published data on the sialome of *Rhipicephalus sanguineus* s.l. (Ixodidae) and *Ornithodoros rostratus* (Argasidae). A partial set of proteins obtained by these studies were used to define the biological process(es) in which proteins are involved. We used a directed network construction in which the nodes are proteins (source) and biological processes (target), separately for the low-level processes (“children”) and the top-level ones (“parents”). We applied the method to feeding *R. sanguineus* at different time slices, and to different organs of *O. rostratus*. The network connects the proteins and the processes with a strength directly proportional to the transcript per millions of each protein. We used PageRank as a measure of the importance of each biological process. As suggested in previous studies, the sialome of unfed *R. sanguineus* express about 30% less biological processes than feeding ticks. Another decrease (25%) is noticed at the middle of the feeding and before detachment. However, top-level processes are deeply affected only at the onset of feeding, demonstrating a redundancy in the feeding. When ixodid-argasid are compared, large differences were observed: they do not share 91% of proteins, but share 90% of the biological processes. However, caution must be observed when examining these results. The hypothesis of different proteins linked to similar biological process(es) in both ticks is an extreme not confirmed in this study. Considering the limitations of this study, carried out with a selected set of proteins, we propose the networks of proteins of sialome linked to their biological processes as a tool aimed to explain the biological processes behind families of proteins.

## Introduction

Ticks are obligate hematophagous parasites that feed on terrestrial vertebrates (Estrada-Peña, 2015). These parasites can transmit a large number of pathogens, including viruses, bacteria and parasites, for example Crimean-Congo hemorrhagic fever virus, the tick-borne encephalitis virus, bacteria of the *Borrelia burgdorferi* s.l group or protozoans like *Babesia* spp (Hoogstraal, 1979; Hasle, 2013). These infective agents cause major diseases in animals and humans. Therefore, ticks are considered a prominent problem in Public Health (de la Fuente et al., 2008; Madison-Antenucci et al., 2020). Ticks are classified in four families, three extant (Argasidae, Ixodidae, Nuttalliellidae) and one extinct (Deinochrotonidae) (Estrada-Peña and de la Fuente, 2014; Peñalver et al., 2017). The majority of ticks belong to Ixodidae or Argasidae, which have numerous different characteristics to exploit different environmental niches and hosts (Hoogstraal, 1979). One of the striking features between Ixodidae and Argasidae is the feeding, defined as the ingestion of blood to complete their biological cycle. This process may last days for Ixodidae but around 60 minutes for most of argasids (Martins et al., 2020).

Salivary glands have an important role in the tick feeding. These organs participate in the dynamics of feeding by producing substances that mediate the feeding process. Nowadays, we know that a pleiad of proteins, known as the sialome, are involved in the regulation of the tick feeding (Ribeiro and Mans, 2020). The sialome is composed of a large array of proteins that enable ticks to modulate the host immune response (Francischetti et al., 2009) but also, acting on hemostatic, cytolytic, inflammatory process, angiogenesis and wound healing, or the production of the cement that allow some ixodids to attach during feeding (Šimo et al., 2017; Villar et al., 2020). Salivary glands are large organs with different acini that produce the sialome and other chemicals. The acini are classified in different types, each one with diverse physiological actions. For example, type I acini has been classically linked to the water intake, preventing dehydration (Needham et al., 1990; Sonenshine, 1991; Gaede and Knülle, 1997). Recent studies confirmed these observations (Kim D. et al., 2016).

The acini type II and III are major saliva producers in hard ticks contributing to the saliva cocktail. Specifically, type III acini are thought to be involved more in fluid production/expulsion while the type II likely produce proteins, lipids and other bioactive molecules (Sonenshine, 1991). Argasid and Ixodid tick salivary glands are composed by different types of acini and cells, resulting in different actions. This may likely contribute to large differences among the proteins and processes of the organs. Molecular techniques helped in the understanding of the tick feeding. Since the first identification and description of proteins in the saliva of *Ornithodoros* spp., *Ornithodoros moubata* or *Ixodes scapularis* (Ribeiro et al., 1985; Waxman and Connolly, 1993; Ribeiro and Mather, 1998) recent studies had largely increased the number of proteins in the tick sialome (Couto et al., 2021; Karim et al., 2021). The common approach has been the identification of each protein (or its orthologs) of species in the genera *Amblyomma* (Tirloni et al., 2017; Karim et al., 2021), *Hyalomma* (Ribeiro et al., 2017), *Haemaphysalis* (Luo et al., 2019; Wang et al., 2019), *Ixodes* (Kotsyfakis et al., 2015; Kim T. K. et al., 2016; Tirloni et al., 2017), *Rhipicephalus* (Yu et al., 2015; de Castro et al., 2017; García et al., 2020; Tirloni et al., 2020; Couto et al., 2021), and *Ornithodoros* (Araujo et al., 2019; Oleaga et al., 2021; Pérez-Sánchez et al., 2021). The list of references is not exhaustive but shows the diversity of studies on the topic. A major event involved in the complexity of the sialome is the co-called “switching sialome” in which ticks switch their proteins (Valenzuela et al., 2002; Karim and Ribeiro, 2015; Perner et al., 2018). It seems that a “switching of proteins” happens at certain periods of time along the tick feeding (Perner et al., 2018). The hypothesis behind this is that these changes could result from the modification in the expression of some individual genes of the same family or paralogs (Ribeiro and Mans, 2020). However, how the “switching of proteins” affects the chain of biological processes of the salivary glands has not been addressed yet.

However, published reports have focused mainly on the identification of proteins, its characterization and classification into families, thus obtaining a growing knowledge of the activity of these proteins. While existing publications explored the functional families of proteins (i.e., Couto et al., 2021; Karim et al., 2021) an overview of the physiological concepts (i.e., biological processes) is lacking. We think that the development of *in silico* tools to evaluate the biological processes occurring in tick salivary glands could be an essential tool to understand the tick feeding. This study aims to develop an approach defining the timeline of the biological processes involved in tick feeding. It is deeply rooted in the graph theory, main methods being already developed to explain the metabolic routes of *Anaplasma phagocytophilum*, a tick-borne pathogen (Estrada-Peña et al., 2018). Tick feeding is a complex and dynamic process. The proposed methodology intends to obtain the functional side of the proteins detected in the salivary glands of ticks during the feeding, and how the biological processes change along time slices. We can consider this study as a partial proof-of-concept. Our results are not the “complete tick salivary physiology” but a detailed proof of what can be achieved as our knowledge in tick’s sialome continues improving.

This study is focused on the description of the sequential changes of biological processes in the feeding of *R. sanguineus* s.l. using methods from graph theory. We aimed to propose a common framework for capturing the cascade of biological processes involved in the tick feeding. This development allows to pinpoint the main differences of the biological processes between feeding ixodids and argasids. We used the annotated datasets on the sialome of *Rhipicephalus sanguineus* s.l. published by Tirloni et al. (2020) and the data on salivary glands and gut of *Ornithodoros rostratus* (Araujo et al., 2019). The first provides a detailed overview of the proteins expressed at different periods of the feeding of *Rhipicephalus sanguineus*. The second permits comparison of the former with a fast feeder argasid. We consider that this perspective allows to examine the feeding process as an entity, enabling the perception of changes of the biological processes.

## Material and Methods

### Background

We present an approach to the study of the biological process in the salivary glands of feeding ticks supported by the robust field of graph theory. The aim of this study is to present how the expression of proteins can be translated into biological processes, linking the partners into a construct known as a network or graph; a secondary focus is to outline the main differences of biological processes in feeding argasids and ixodids. Our purpose is to demonstrate the basis of a solid comparison of the changes of biological processes along the feeding period of a tick. The study is based on recent and high standard datasets, one of them referring to *R. sanguineus* s.l. (Tirloni et al., 2020) the other to *O. rostratus* (Araujo et al., 2019). After a preliminary filtering (see below) processes were obtained through queries to online repositories. To handle this data, we did use the graph theory framework, which establishes links between protein(s) and process(es). Therefore, significant indexes can be derived from the network. Since we obtained data for the two major families of ticks, we contrasted the differences in feeding types.

### Collection, Screening, and Re-Assessing Protein Datasets: Capturing the Biological Process

We conducted a literature search in public repositories, like Pubmed, Google scholar and Scopus following the PRISMA statements (Moher et al., 2009). In the compilation we selected those studies that were found under the search terms: (“tick(s)” OR Ixodidae OR Argasidae) AND (“salivary glands” OR “gut”) AND (“sialome” OR “sialotranscriptome” OR “proteome”) with special attention to the **Supplementary Material** of each study. The query produced a relatively low number of published papers; therefore, we also evaluated the bibliography of each paper to complete the dataset. Data was selected to contain some details, as follows: (I) the name of the protein had to be following standards in SWISS-Prot/Uniprot KB database for improving further queries (see below); (II) the protein reads had to be in TPM (transcripts per million); (III) the data should refer to “slices” of the feeding process (in ixodids); (IV) we aimed to include contemporaneous studies on argasid ticks. Aiming to comparison, it is important that the studies were carried out at similar periods of time, since fast pacing advances in these techniques may turn results matchless. Two studies fulfilled these standards, one of them dealing with *Rhipicephalus sanguineus* s.l., (Rs) and the other with *Ornithodoros rostratu*s (Or). In the study dealing with Rs (Tirloni et al., 2020) data is partitioned according to the feeding moment using the weight of the tick, which is a better estimation than the elapsed time after the onset of feeding. The study determined seven feeding groups: unfed ticks; group 1 (G1) with an average weight of 1.8 mg, collected at day 2 of feeding; group 2 (G2), averaging 3.6 mg, collected at day 6 of feeding; group 3 (G3) averaging 7.0 mg, collected also at day 6 of feeding; group G4 (G4) averaging 10.9 mg, collected at day 8 of feeding; group G5 (G5) averaging 24 mg, collected at days 8 and 11 of feeding and group 6 (G6) averaging 36 mg, collected at days 6, 10 and 13 of feeding. The Or study (Araujo et al., 2019) is aimed to compare the proteins in both salivary glands and gut, considering the short feeding time of soft ticks. This selection did not want to exclude other datasets because its unreliability, but because the selected ones correctly matched the aims for developing our framework.

We performed a data cleaning of both datasets, including only proteins in the original spreadsheet about the complete transcriptome of the **Supplementary Material** by Tirloni et al., 2020, with a coverage equal or higher than 90% (column BM), an E value equal or less than 10^-5^ (column BI) and a similarity equal or higher than 50% (column BL). A similar filtering was followed for Or, with the same parameters, using columns FT, FZ, and GA, respectively, of the original datasheet by Araujo et al. (2019). To note that a selection of proteins restricts their number, and therefore affects conclusions. Even more, both datasheets represent different experiments. The proteins fulfilling these criteria were queried to UniprotKB (UniProt Consortium, 2021). Uniprot is a collection of annotated proteins that is manually curated and constantly updated. We search the actual name of protein given by the original datasheets for Rs and Or, without performing a BLAST query, since the original datasheets contain these names. In short, the list of proteins to be queried was uploaded to Uniprot, using the “Retrieve/ID mapping” option, and marking the option “biological processes” in the selection of downloadable columns. This returned the set of biological process(es) in which each protein is involved, including the Gene Ontology value(s) associated to each protein (GO; Ashburner et al., 2000; Gene Ontology Consortium, 2021). GO is a database that assumes that if a gene has the same behavior as other in a specific category of gene ontology, the gene belongs to the same category. Not every protein had an annotation; a few proteins were therefore impossible to ascribe to any biological process; these proteins (less than 1%) were dropped from the study. All the biological processes have its own GO term associated number, from the most specific biological process to the most general one, which is also returned as a “cascade” of processes by Uniprot. This allows to correlate “child” and “parent” processes in the chain of events by GO (i.e., “DNA repair” is a part of “Metabolism”, but other child processes are also part of “Metabolism”; therefore a network approach makes sense, see point 2.3 below). We did a distinction between low-level (LL) processes (the “children”) and top-level (TL) processes (the “parents”). To obtain the complete chain of processes involved in the expression of every single protein we used the package “GO.db” (Carlson, 2019) for the R programming environment (R Core Team, 2020) that reads and handles the GO terms returned by Uniprot for each protein.

### Generating the Networks of Biological Process

A network is a set of nodes that interact through links. Networks can be used to represent i.e., food webs (Ings et al., 2009) associations of parasites with hosts (Krasnov et al., 2012) or gene activations (Smolen et al., 2000). Each link has a property called “weight” that is proportional to the strength of the interaction between any two members of the network. In our application, the weight is the TPM of each protein linked to a process; this is a directed network (i.e., protein-process), but a protein may take part of several processes. The framework is based on the background by Estrada-Peña et al. (2018) analyzing the metabolic processes of *Anaplasma phagocytophilum* growing in human cells.

The power of a network is its ability to obtain indexes that define the importance of each node, either protein(s) or process(es). There is wide variety of indexes that represent several features of the network allowing the evaluation of the main properties of either the network or the individual nodes. Our application measures the importance of each biological process throughout the feeding process or compares biological processes in different species of ticks. We selected the so-called PageRank (PR; Senanayake et al., 2015) that measures the connectivity of a node in reference to the nodes in the network to which it is attached. In other words, a process linked only to one highly expressed protein, will have a different PR than a process linked to many proteins slightly expressed. It is measurement of the “popularity” of the process. In other terms, PR indicates means how “cool” is the biological process as linked to proteins.

**Figure 1:**
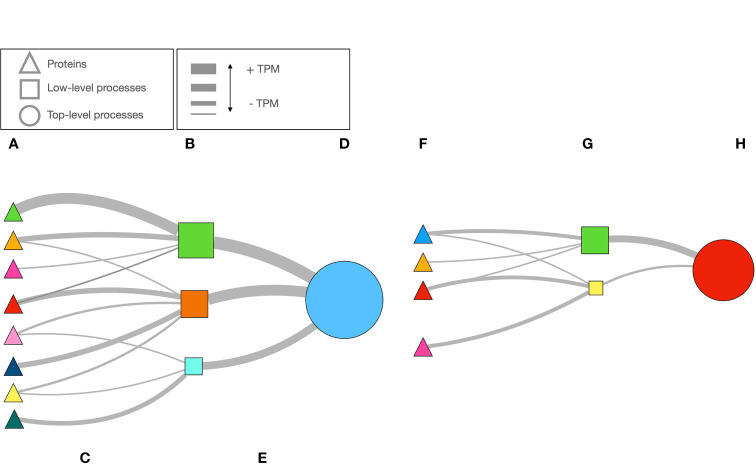
A schematic example of how the network is build using data of the expression of proteins, and how the importance of each biological process is calculated. Once the proteins are selected using very astringent criteria **(A)**, public databases are queried to obtain the biological processes in which they take part **(B)**. This is what we call “low-level” biological processes (e.g., the “children processes”). The strength of the link between the protein and the process is the TPM of the protein, that we schematically show as the width of the line **(C)**. With the TPM, we calculated the PageRank index for each process in **(B)**. We schematically shown the PageRank as different sizes of the squares in **(B)**. Each low-level process (i.e.,“fermentation of sucrose”) is part of a cascade of biological processes, of which we identified the top ones in the hierarchical chain (i.e., “metabolism”, the “parent” process”). From the sum of TPM’s of the proteins impacting each low-level process, as before, the width of the lines **(E)** represent the strength of link. The PageRank value of the top-level process **(D)** is calculated. However, if the number of proteins or their TPM are low **(F)** the PageRank of the chain of processes will be lower **(G, H)**. The opposite result (not illustrated) would happen in the contrary case (more proteins expressed or with higher TPM). PageRank is a very valuable index to measure the “popularity” or the “coolness” of a process, and therefore an explicit statistical description of its importance in the complete metabolical chain studied.

**Figure 2:**
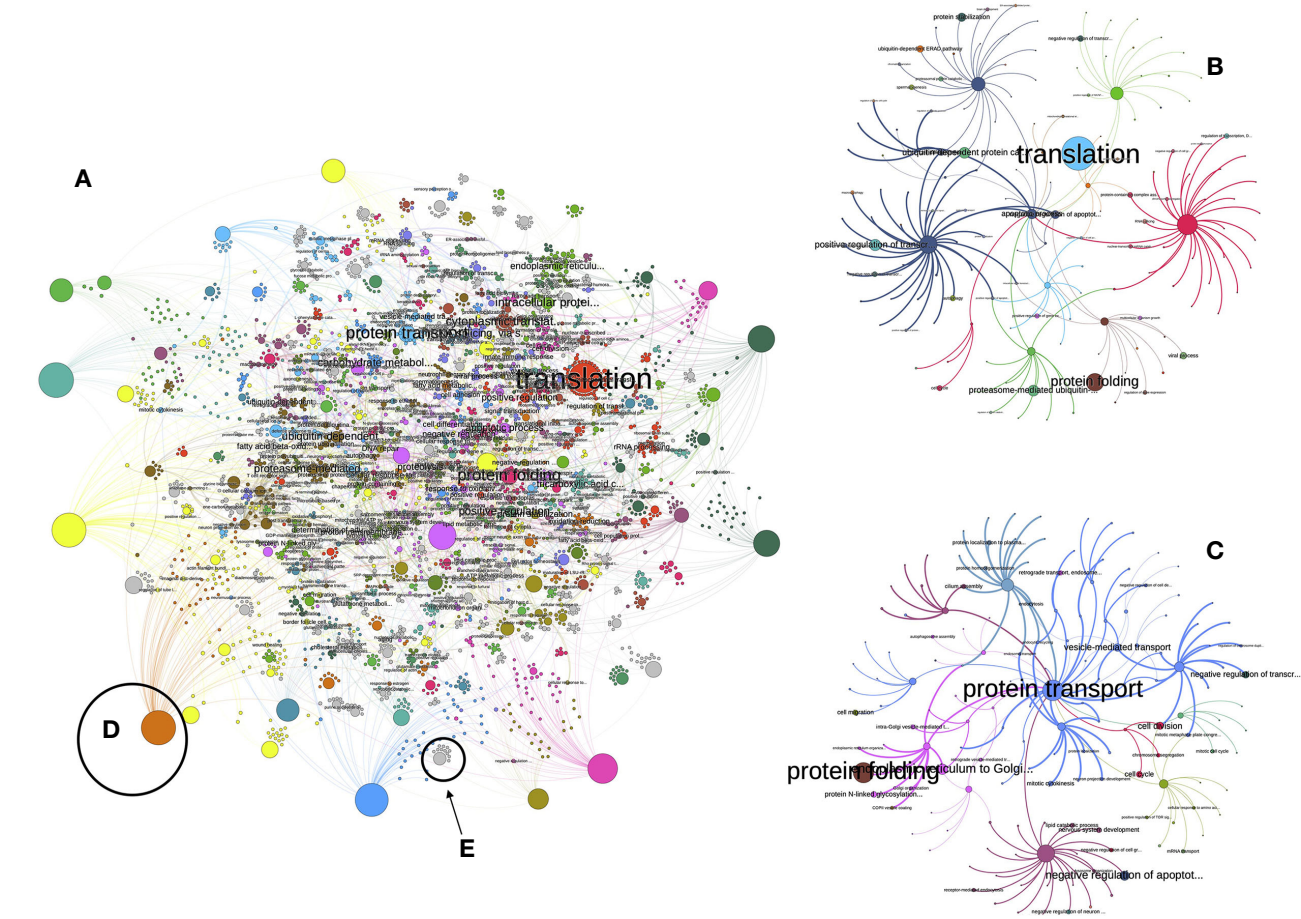
**(A)** The view of the network of low-level processes of the unfed *R. sanguineus* ticks. The figure is not intended to follow every single link but is just a visual depiction of the enormous complexity of that network. Each circle is either a protein or a process. Only the processes with a PageRank higher than 1 are labelled. Colors mean for clusters, or groups of proteins and processes that tend to appear together more frequently than with others. The size of each circle is proportional to its PageRank (some large unlabelled circles are proteins). In both **(B, C)**, we plotted the subnetworks related to the “translation” and “protein folding” processes, aiming to improve the visualization of how the network is build, with the same conventions of colors and sizes. More examples are illustrated in **(D)** (in which a very prominent protein is linked with many processes, because many links start in it) or in **(E)** (in which a low-importance process of medium size is linked to a few proteins involved in the process, with low importance and smaller size).

**Figure 1** sketches the explanation above. There are proteins (**Figure 1A**) with different TPM’s linking to low level biological processes (**Figure 1B**). The width of the lines is proportional to the protein’s TPM (**Figure 1C**). The LL processes are the “children” of TL processes (**Figure 1D**). These LL or TL processes are provided by the hierarchy of GO, e.g., a LL process (like “activation of C-type lectin signaling”) is part of a TL process (in the example, “response to stress”). It may happen that some proteins are missing (**Figure 1F**) or their TPM’s are higher or lower; therefore, the PR of both LL and TL will change (**Figures 1G, H**, schematized according to the width of lines and the size of the circles). The PR index is thus a way to measure the expression of a biological process. **Figure 2** includes an actual network of the LL in Rs G1 feeding ticks, as well as some sections augmented to provide with better context to the readers. The purpose of the **Figure 2** is merely demonstrative and is not intended for the visual observation of each individual node or link.

We generated separate networks for either the LL or TL in the case of the salivary glands of Rs for each period of engorgement, as well as for salivary glands and gut of Or. The PR was calculated for each network. All the networks were built using NodeXL (https://nodexl.com; last accessed, June 2020) and Gephi v0.92 (Bastian et al., 2009). The purpose was to compare the different PR of LL and TL in the feeding timeline of Rs, and to pinpoint the main differences with Or. We will refer to “unfed” or “G1” to “G6” the periods for Rs (according to the original definitions by Tirloni et al., 2020), and “OrSG” or “OrGut” to the corresponding salivary glands or gut of Or.

### Analysis of Data Using Statistics Describing of the Processes Involved in Feeding

We wanted to estimate the variability of the PR of each LL or TL along the feeding period of Rs. We employed the percent variance of the PR as a reliable statistical analysis: the value of PR in each group gives the percent variance with respect to the past value along consecutive feeding groups. This procedure produced information for literally thousands of LL, which resulted inadequate for a graphical representation (see **Supplementary Table 1** for the complete list of LL used in this study). Therefore, after the calculation of the percent variance between any two consecutive groups, we selected the LL that have a variance of change above the percentile 98 (i.e., the top 2% of higher variability) providing a total of 71 LL that display the maximum rates of change throughout the feeding period. As explained before, our aim is not to analytically examine every single process, but to provide with examples about the network application and its possibilities. We however included the complete set of TL and charted the variability of PR. Charts plotting values of PR were generated using the R environmental programming (R Core Team, 2020). The number of shared proteins or LL among feeding groups and species was compared using Venn diagrams (http://bioinformatics.psb.ugent.be/webtools/Venn/, last accessed August 2021). The topology of Venn diagrams does not allow to use more than 5 different groups in one single chart; we therefore produced these charts separately into meaningful groups.

We aimed to demonstrate the usefulness of the method selecting one of the TL that did show changes along the feeding of Rs, namely “response to stress”. We chose every LL involved in “response to stress”, according to the hierarchy in GO, and produced a heatmap of the PR values of the top LL showing the maximum rates of change (about 30% of the total). We also obtained subnetworks involving only the selected LL’s. To note, this is not a purposely selected chain of processes aimed to demonstrate a particular event. It is intended only as a proof-of-concept of the reliability of PR to selectively track the changes of biological processes in the metabolism of the tick and to know the processes of the salivary glands *via* the relative composition of the sialome. It must be noted, too, that because we did a selection of proteins, driving to a selection of the processes under change, the results obtained cannot be interpreted as the *complete* sequence of changes through feeding, but a demonstration of the study flow.

The discussion of this study includes possible gaps that this approach could produce on the results obtained, mainly regarding the filtering proteins and the hypothesized problems derived from the original BLAST hit. With the actual knowledge and limitations in the field, we consider studies of this kind will move forward with the improvement of annotations of proteins in ticks.

## Results

### General Results

Although this study is not devoted to the proteins identified in the sialome of both species of ticks, we think a background is necessary for further presentation of results. Some of these results have been already published in the original papers and are herein provided aiming to completeness. We describe first the general results about the number of proteins and LL in Rs, then we focus on the LL and TL of Rs. We will briefly describe the results for Or and compare between Rs; last, we demonstrate the framework with an example made on data about “response to stress” in Rs. As mentioned later, some caution should be exerted reading the Results, mainly regarding to the preliminary selection of proteins of the study.

**Figure 3:**
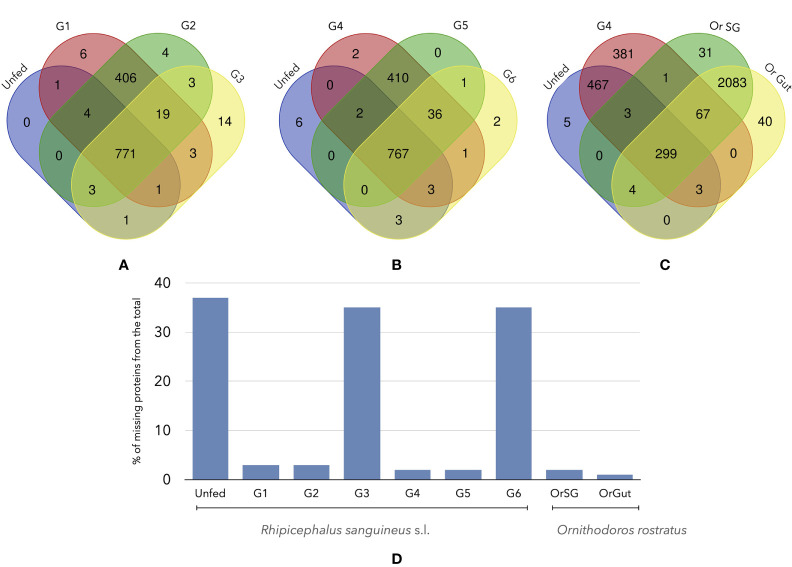
Venn diagrams of the number of shared proteins among different groups of unfed or fed *R. sanguineus* ticks and/or *O. rostratus*. **(A)** *R. sanguineus* unfed, G1, G2, and G3; **(B)** *R. sanguineus* unfed, G4, G5, and G6; **(C)** *R. sanguineus* unfed, G4, and *O. rostratus* salivary glands or gut. The terminology of groups of feeding *R. sanguineus* follows Tirloni et al. (2020). The intersection of each diagram shows the number of shared proteins between these specific groups. **(D)** Histogram show the quantity of proteins that are non-present of the total of each tick specie.

After cleaning of the datasets of proteins, we obtained a set of 1166 proteins in Rs (all groups) and 2150 in Or (SG and Gut). Some of these proteins are shared among the different groups of Rs and with Or, but in different proportions regarding the species and the feeding groups (**Figure 3**). In the case of Rs, there is a set of core proteins (767-771, **Figures 3A, B**) that are always present in all the groups. It is important to remark some interesting associations: (i) the high number of shared proteins between G1-G2 (406, **Figure 1A**) and G4-G5 (410, **Figure 1B**) but not G6 (36, **Figure 1B**) (ii) the fewer shared proteins between Or and the groups of Rs (299, **Figure 1C**) (iii) the high similarity of the proteins detected in both SG and guts of Or (2083, **Figure 1C**); only 31 proteins have been detected solely in SG of Or, and 40 in its gut. In summary (**Figure 1D**) unfed Rs lack 464 proteins already found (according to our filtering rules) in other feeding pools; groups G3 and G6 of Rs lack 430 and 432 proteins, respectively, that were detected in the other groups. The maximum number of non-shared proteins among the groups of Rs is six (in a total of 1246). Collectively, Rs and Or do not share the 91% of the total proteins recorded in both species (but read “Discussion” about the possible gaps in the design of the calculations).

**Figure 4:**
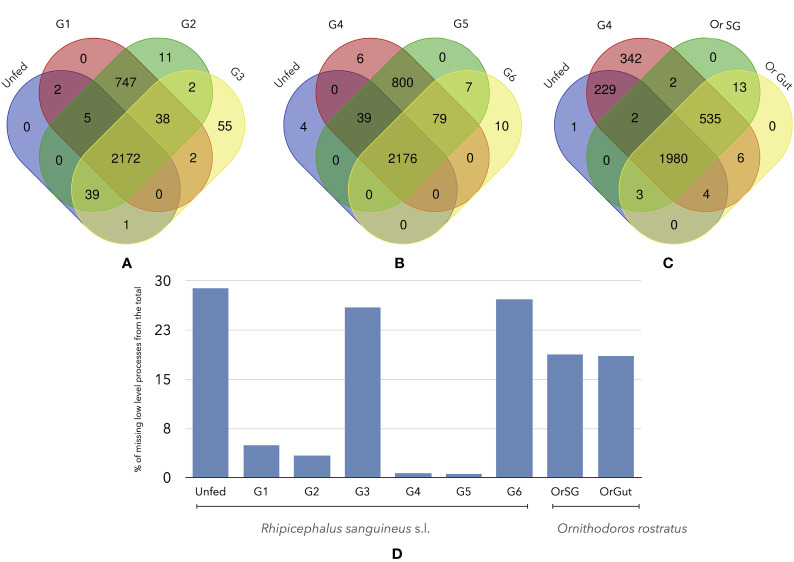
Venn diagrams of the number of shared LL processes among different groups of unfed or fed *R. sanguineus* ticks and/*or O. rostratus*. **(A)** *R. sanguineus* unfed, G1, G2, and G3; **(B)** *R. sanguineus* unfed, G4, G5, and G6; **(C)** *R. sanguineus* unfed, G4, and *O. rostratus* salivary glands or gut. The terminology of groups of feeding *R. sanguineus* follows Tirloni et al. (2020). The intersection of each diagram shows the number of shared proteins between these specific groups. **(D)** Histogram show the quantity of processes that are non-present of the total of each tick specie.

The queries to Uniprot and GO produced a variable number of LL (**Figure 4**). There is a total of 3121 LL in Rs, considering every feeding group; Or has 2535 LL in the SG and 2541 in the gut (2531 of them are shared). The core group of LL processes shared by Rs is 2172-2176. However, when compared against Or, the number of shared LL drops to 1980 comparing the unfed group with fed Or. Also, this number continued to fall to 535 when comparing a fed group of Rs and feeding Or. This is a striking result, since the lack of 91% of shared proteins between Rs and Or drives the lack of only the 10% of LL biological processes. These results should be observed with caution. Up to 902 LL (collectively detected in the feeding groups) are missing in unfed Rs. Of interest, both G3 and G6 feeding Rs lack 812 and 849 LL.

### The “Low Level” Processes of *R. sanguineus*

**Figure 5:**
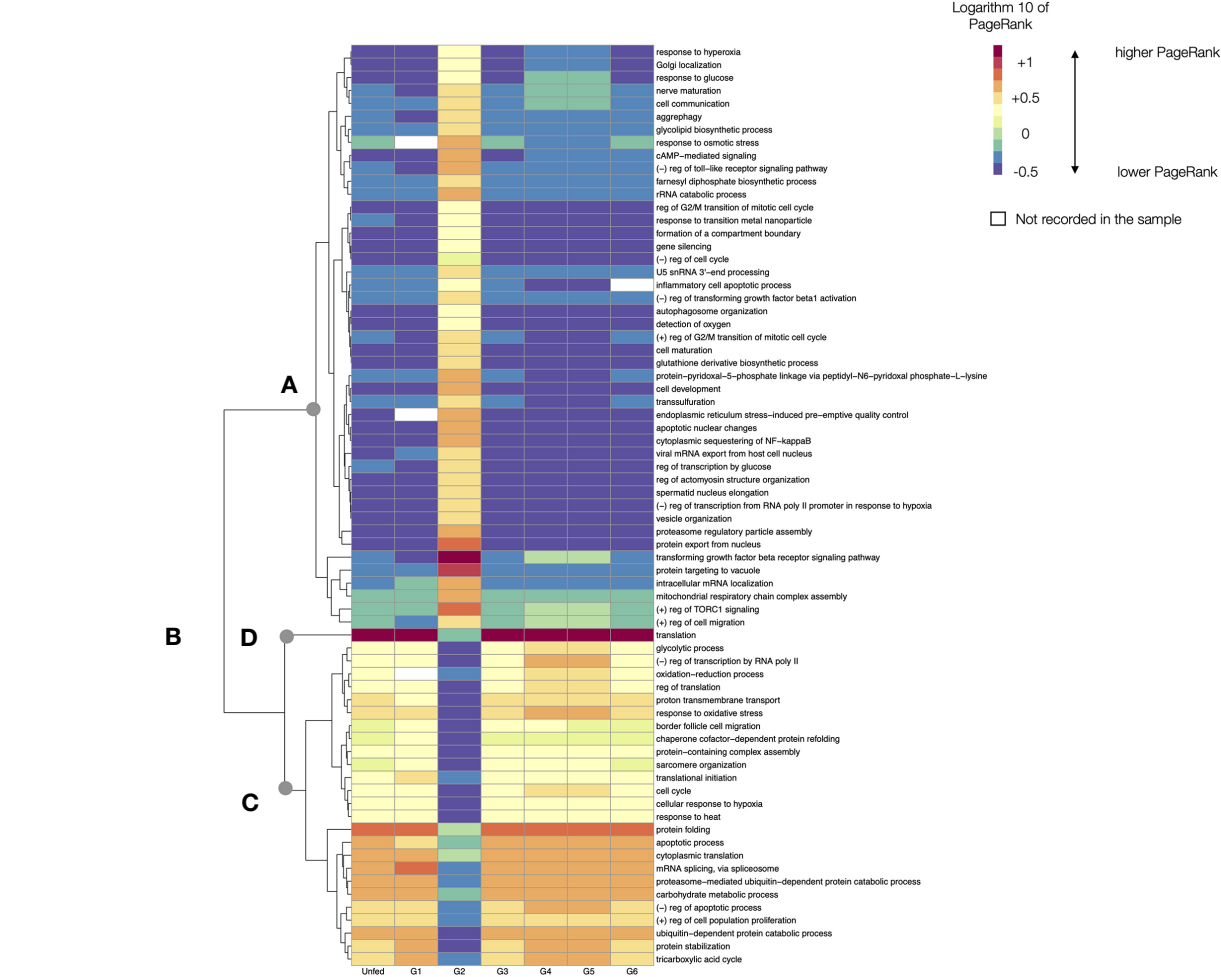
A heatmap displaying the values of the log10 of PageRank of the low-level processes in unfed and feeding R. sanguineus. The chart displays only the processes with a rate of change located in the top percentile 98% of variability (i.e., only the 2% of processes, those with highest variability, are shown). The heatmap shows the processes grouped according to a dendrogram (left) grouping those processes with similar patterns of variability. Dots labelled **(A–D)** show breaking points of the dendrogram, intended only as a help for interpretation. The tick group (unfed, G1 to G6) is included at the bottom of the figure, following the terminology of the original data by Tirloni et al. (2020). The name of the LL process is at right of the chart. In the legend, the symbol “+” denotes “positive” and the symbol “-”denotes negative. Cells in white are absent processes.

The LL represent the most fundamental bricks of the biological processes of the feeding tick. A chart of the changes of PR of each LL through the feeding sequence of Rs would be hardly readable. We thus chose the top 2% of most variable LL’s charting the log10 of their PR values. The heatmap in **Figure 5** includes a dendrogram grouping the LL that tend to have similar values of PR along the tick feeding. The analysis of the LL processes in the top 2% reveals important differences in the functional biology of groups of feeding ticks. These LL can be grouped into three categories: those that have a relatively low PR (but in G2), those that keep a high PR throughout the feeding process, like “translation” (but in G2) and those that show intermediate to high values of PR, with slight variations through the whole feeding (but in G2). To note, the heatmap shows only the top 2% of LL with highest rates of change, obtained from an already filtered subset of proteins of the total proteome.

Without questions, G2 represents a special breakthrough point in the feeding process of Rs at the LL observational level, as it could be expected for ticks beginning the feeding process. The analysis shows that some LL are suddenly activated at G2 (breakpoint “A” in **Figure 5**), while some others decrease its PR, even if they keep the highest values in other groups (like “translation”, breakpoint “B” in **Figure 4**); others are “arrested” specifically at G2, even if they are expressed at medium (breakpoint “C” in **Figure 5**) or higher values (breakpoint “D” in **Figure 5**) in the rest of feeding groups. As mentioned, the figure is intended to show the analytical abilities of a network approach to extract conclusions using only the TPM of the detected proteins.

### The “Top Level” Processes of *R. sanguineus*

The analysis of TL processes narrates a different history of the feeding Rs. We identified 47 TL processes in unfed and feeding Rs. The networks produced for the TL for all the groups are provided as part of the **Supplementary Material Figures (S1–S7)**. Interestingly, some TL processes remained undetected at some time slices of the feeding of Rs. The analysis of TL clearly states that major changes in the sialome of Rs are obviously produced at the beginning of feeding (G1) in which many TL increase its activity. This finding is not contradictory with the previous comments about LL. We consider that the analysis of TL is a “panoramic view” of the biological processes of the SG of the tick, while LL are the bricks of the chain of events of the tick feeding. The number of LL per TL is very variable. Some TL are represented by only a few LL (or even only one). This immediately translates into the very variable number of proteins that are involved in the set of LL.

**Figure 6:**
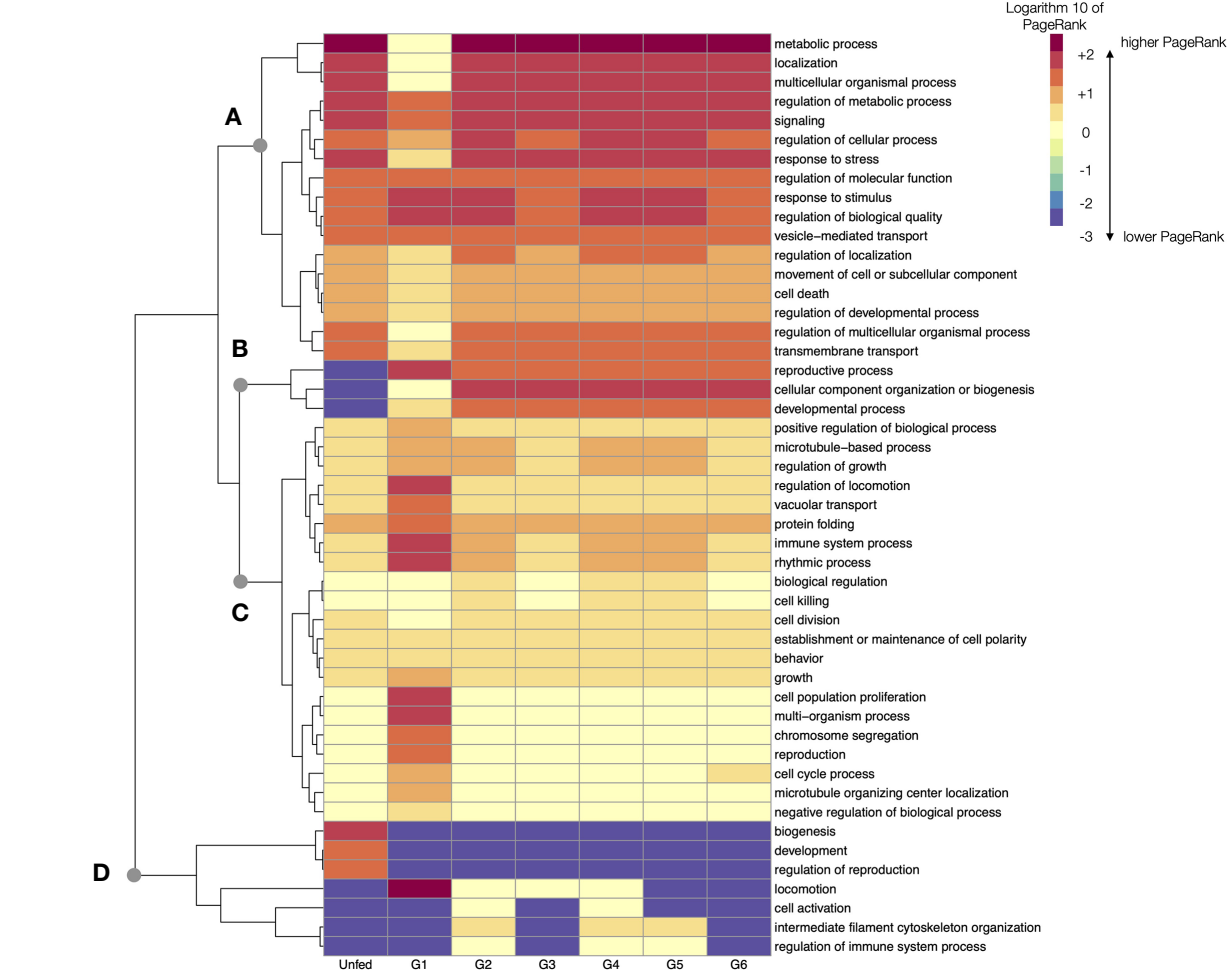
A heatmap displaying the values of the log10 of PageRank of every top-level processes in unfed and feeding *R. sanguineus*. The heatmap shows the processes grouped according to a dendrogram (left) grouping those processes with similar patterns of variability. Dots labelled **(A–D)** show breaking points of the dendrogram, intended only as a help for interpretation. The tick group (unfed, G1 to G6) is included at the bottom of the figure, following the terminology of the original data by Tirloni et al. (2020).

The **Figure 6** charts the log10 of PR of every TL in the consecutive feeding groups of Rs. This figure re-arranges them to produce a dendrogram of the TL that change its PR in similar patterns along the feeding. The beginning of feeding (G1) marks the expected breakpoint in comparison with the unfed ticks. The pool of G1 have the most notorious changes of PR in more than 80% of TL processes. Some TL experience a decrease of PR from unfed to G1, and then continue to be highly represented in ongoing groups (breakpoint A in **Figure 6**). Exceptions are “reproductive process”, “biogenesis”, and “developmental processes”, all of which have a low PR that increases in G1 and continue at high values until the termination of feeding (breakpoint B in **Figure 6**). To note that the TL associated to reproduction show a clear increase as soon as G1, and not at the end of the of feeding: the morphological and physiological changes associated with the reproduction start as soon as the tick begins to feed. Some other TL experience a relative increase in G1 to decrease again in the subsequent groups (breakpoint C in **Figure 6**).

In terms of absolute rates of change of PR between unfed and G1, the PR of “cell proliferation” increased more than 65,000 times, “localization” (a TL involving the cellular organelles) increased more than 13,000 times and the “immune system processes” increased almost 2,000 times. All these TL returned to levels comparable with unfed ticks after G2. However, ticks in G2 group experienced dramatic changes, mainly those related to “metabolic processes” (with a PR 54,000 times higher). Most other TL processes have lower changes through the feeding. The changes occurring in the G6 group that includes ticks detached after feeding are of interest. In example, the “metabolic process” that remained at relatively low levels through the previous phases of the feeding period (but for G1) increase again more than 9,000 times, overlapping with the final period of intense blood intake.

The changes observed in LL have not a direct translation to TL; they are not directly correlated, since i.e., most prominent changes of LL were noticed in G2. We hypothesize that there are two levels of observation: the LL allow to capture a finely tuned description of the actions of tick sialome, while TL only allow a wide (but reliable) description of changes. Anyway, even if TL alone may be a rough way to define the physiology of the feeding ticks, regarding the fine detail obtained examining LL, the changes in tick sialome through feeding are obvious and have a biological support.

### *Ornithodoros rostratus* and the Example of a Fast Feeder

**Figure 7:**
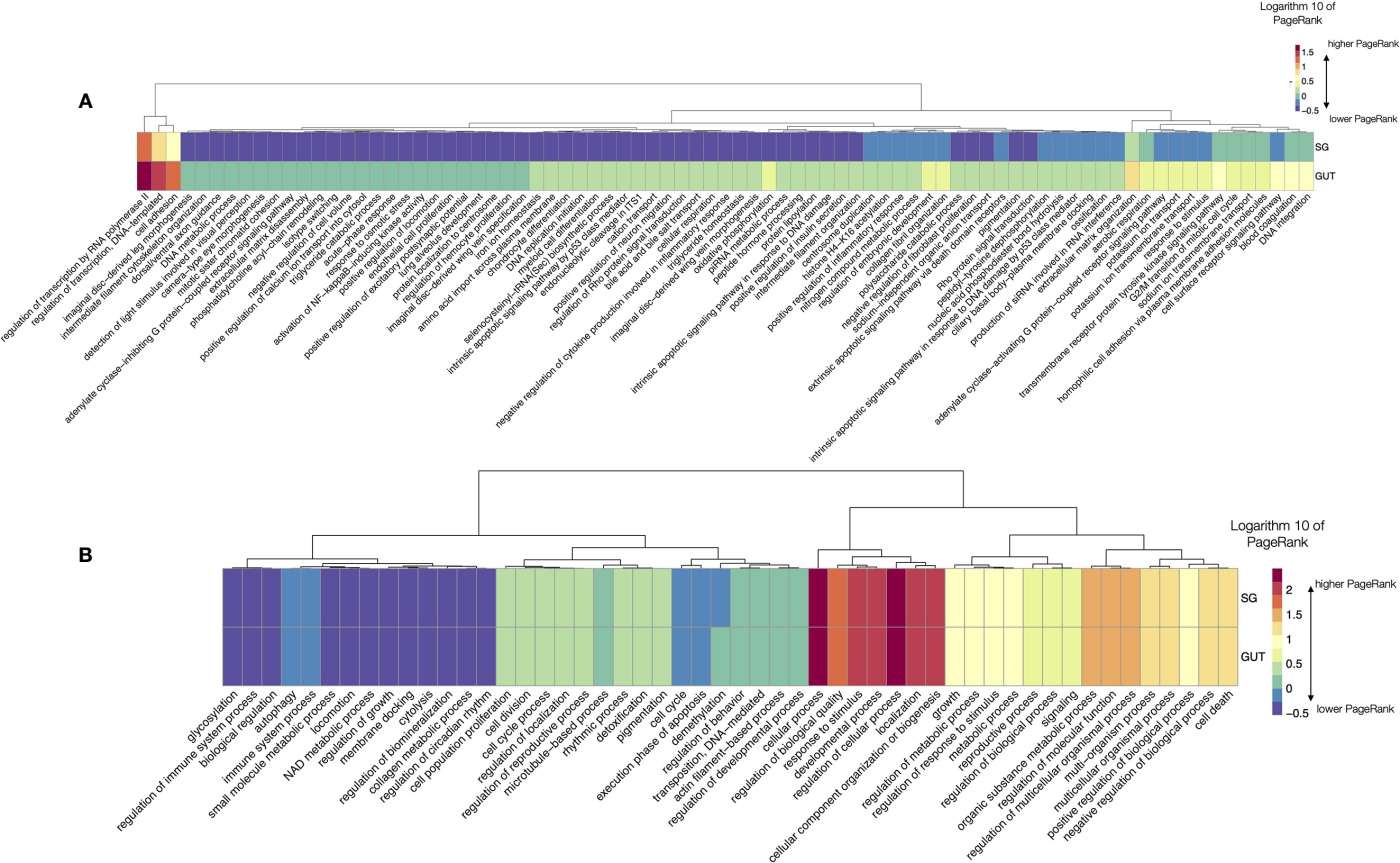
**(A)** A heatmap displaying the values of the log10 of PageRank of the low-level processes in salivary glands (SG) and gut of *O. rostratus*. The chart displays only the processes with a rate of change between organs located in the top percentile 99% of variability (i.e., only the 1% of processes, those with highest variability, are shown). The heatmap shows the processes grouped according to a dendrogram (top) grouping those processes with similar patterns of variability. Dots show breaking points of the dendrogram, intended only as a help for interpretation. **(B)** A heatmap displaying the values of the log10 of PageRank of the top-level processes in salivary glands (SG) and gut of *O. rostratus*.

This study is mostly focused to capture the dynamics of the changes of sialome in the feeding *R. sanguineus*. However, we aimed to compare data for two species of the major families of ticks, using data of Or as an example. To note, the dataset of Or only reported one slice of time, and is not extended into the process of digestion. Also, the dataset of Rs lacks data on gut, so the comparison is limited. As before, we did chart the LL processes that have significantly different log10 PR in SG and gut (**Figure 7A**) and the complete set of TL processes (**Figure 7B**). Of interest, most variable LL have higher PR in the gut than in SG. Variability in the remaining LL (more than 2000 LL) is lower. **Figure 7B** displays the log10 of PR of the complete set of TL in both organs. Differences are minimal and there is an almost pair-to-pair correspondence of the PR values of TL in the examined organs of Or. The networks produced for the TL for both organs are provided as part of the Supplementary material Figures (S8, S9). Of interest, metabolism related TL have far smaller PR in Or than in Rs, while the cellular processes and a wide array of TL involved in several types of regulation are more abundant.

However, these results, together with those comparing the number of shared proteins in both families of ticks are striking. These results could be derived from an unreliable selection of proteins, since they were extracted and queried from different proteomes. We will further discuss about these findings and the possible sources of error.

### A Proof-of-Concept: Response to Stress in *R. sanguineus*

**Figure 8:**
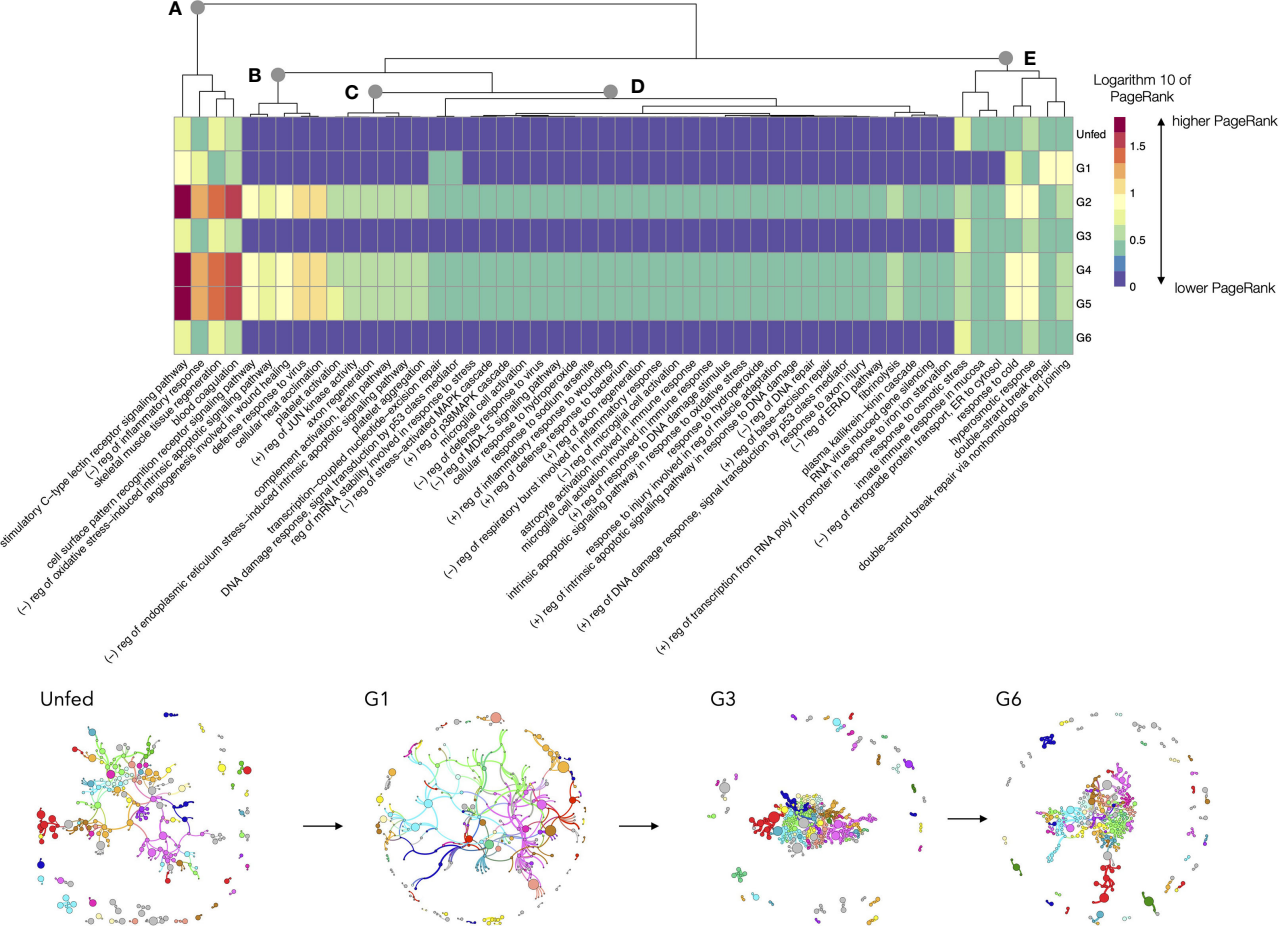
A heatmap displaying the values of the log10 of PageRank of the low-level processes in the subnetwork of processes linked with “response to stress” in *R. sanguineus*. The chart displays only the processes with a rate of change between organs located in the top percentile 70% of variability (i.e., only the 30% of processes, those with highest variability, are shown). The heatmap shows the processes grouped according to a dendrogram (top) grouping those processes with similar patterns of variability. Dots labelled **(A–E)** show breaking points of the dendrogram, intended only as a help for interpretation. The tick group (unfed, G1 to G6) is included at the bottom of the figure, following the terminology of the original data by Tirloni et al. (2020). Subnetworks of proteins and processes for selected groups are included at bottom to show its topology. Colors mean for modularity, or groups of proteins and processes that tend to appear linked significantly more frequently than with others. The size of each circle is portion to its PageRank and the width of the lines denote the strength of association. To note the cohesively linked dots in ticks belonging to G1. However, G3 and G6 show a large group of dots tightly located in the center of the network, denoting large interactions among them, while some of the proteins and processes that do not belong to the nucleus and are located in the periphery.

We focused on the analysis of the chain of events involved in the TL “response to stress” in unfed and feeding Rs. The TL includes a total of 226 proteins and 149 LL. As before, it is not possible to produce a readable chart for every LL; we therefore opted for focusing the analysis on the LL with highest rate of change across the feeding, resulting in the 30% of LL. The **Figure 8** includes a heatmap charting the log10 transformed values of PR throughout the feeding period of Rs including only the LL for “response to stress”. The chart includes a dendrogram grouping the LL that tend to change the PR values in a similar pattern. A major increase of PR is observed for tick pools collected at G2, G4 and G5 (breakpoint A in **Figure 8**). They share the large increase of PR, in LL, like the C-type lectin receptor signaling pathway, the negative regulation of inflammatory response and the blood coagulation. There are LL that increase its PR but at are almost absent at some periods of feeding like i.e., the negative regulation of oxidative stress, the acclimation of cells to heat, and the defense response to viruses (breakpoints B, C in **Figure 8**). Other LL (**Figure 8D**) have relatively medium to low values of PR throughout the complete feeding period, showing some increase mainly at G2, G4 and G5 (i.e., response to cold, or hyperosmotic response) or G1 (processes involved in double strand DNA repair). The rest of the processes (**Figure 8E**) are predominantly common in unfed, G1, G3 and G6, with a small increase in the rest of the feeding phases. Other LL involved in “response to stress” are well below the percentile 70 of rate of change, and they exhibit very small changes through the complete feeding.

The subnetworks obtained for that specific chain of biological process are also included in **Figure 8**, only for some slices of the feeding. The topology of these networks, consisting in a relatively dense nucleus with many dots in the periphery explains the timeline of the process. Unfed ticks have a relaxed structure of proteins, to move into a cohesively linked network of LL in ticks belonging to G1. However, G3 and G6 show a large group of dots tightly located in the center of the network, denoting large interactions among them, while some of the proteins and processes do not belong to that nucleus of the network and are located in the periphery, playing secondary actions.

## Discussion

We applied a previously developed framework, based on directed networks linking proteins and biological processes applied to the metabolome of *A. phagocytophilum* infecting human cells (Estrada-Peña et al., 2018) to the growing datasets of tick’s sialome. In the current study we used previously published data on the proteomics of saliva and gut of two species of ticks (Araújo et al., 2019; Tirloni et al., 2020). Our main aim was to elaborate about a framework that could “convert” the current knowledge about lists/families of proteins into the changes of biological processes of tick salivary glands. Along the tick feeding and to compare the (dis)similarity of these processes between the two major tick families. Our aim is not to repeat the classification of proteins or the role of different families of proteins, as it is already published. This study is not dedicated to explaining, step by step, the physiology of the salivary glands over the grounds of modern “omics” technologies, but to propose a context for a foreseeable approach. Some of our conclusions have been unquestionably demonstrated in other reports, like the changes in salivary glands observed at different times of feeding (i.e., Sonenshine, 1991). We did not intend to replace previous, solid backgrounds on tick physiology, but to propose a framework to analyze the biological processes that emanate from a set of proteins. We aimed to rise the current classification of families of proteins by its involvement in different biological processes.

Several gaps should be considered in our study. We must stress the limitations of this approximation because we used two different datasheets, for two different ticks and different research groups. Also, it is necessary to indicate that we obtained partial representations of the proteome of either salivary glands or gut of both species, filtering the original data on proteome aiming to obtain the best characterized proteins. It could be possible that the same selection rules for both datasheets produced a different list of proteins, even if they are present but with a slightly lower value than our filters. Since the processes was obtained from the denomination of the protein, the results may be influenced by the selection did over the original set of proteins. For example, the coverage is indicative of the assembled length of the contigs and may be transcriptome dependent, the E-value <10^-5^ would probably capture housekeeping proteins, and the similarity may be lineage dependent, so that a soft tick gene may give a similarity of 48% while a hard tick gene may give a similarity of 51%. In addition, TL are redundant because different pathways may drive to the same high-level processes. Therefore, misidentifications of the correct LL, would drive to unexpected variability of the basic results, while slightly impacting the TL. The comments above are of special application to the comparison between the two tick species. This study could not represent the complete set of biological functions, but it could be interpret as proof of concept for a methodology in proteomic tick field to show biological function behind the transcriptome of the proteins.

We agree with Ribeiro and Mans (2020) that we are far from knowing the complete sialome of ticks; therefore, the current exercise is aimed to encourage further developments to present the results of transcriptomic analyses. We deliberately chose published datasets in which explicit mention of the E-value, the % of coverage and the expression of each protein expressed as TPM were included. It has been demonstrated that TPM is the best representation of the “amount” of the protein present in the sample (Wagner et al., 2012). These datasets contain either sequential estimations of the sialome through the (long) feeding of an ixodid, like Rs, or the proteins present in SG or gut of a fast feeder, like Or. We are aware that some proteins may be erroneously identified because the lack of a complete coverage of the tick sialome, therefore biasing the results. The logic behind our results confirm that other than spurious unreliabilities, the translation of proteins into biological processes is reliable within some limits and that the panorama (even if partial, as is the case) carries a solid message. Previous efforts to classify proteins according to functions (i.e., Wang et al., 2019) provided relevant information on tick sialome, and explored interactions between proteins but did not observe the whole set of interactions driving protein(s) into process(es).

A complete description of the morphological features of the tick’s salivary glands was compiled by Sonenshine (1991). The author summarized his own and previous observations by other authors about the morphological changes of the organ, because of the different steps of the feeding process. Sonenshine (1991) already correlated these changes with the secretion of the few molecules that could be detected by the technology of the time. These morphological changes are reflected in the dynamics of the production of saliva, regarded as an expression of the different phases of ixodid tick feeding. The accessibility of methods allowing mass detection and expression of proteins did open a new field and supported previous findings based on the microscopical examination of the salivary glands. Using different technologies, the study of the sialome has been addressed in numerous species of ticks (i.e., Kotsyfakis et al., 2015; Yu et al., 2015; Kim T. K. et al., 2016; de Castro et al., 2017; Ribeiro et al., 2017; Tirloni et al., 2017; Tirloni et al., 2017; Luo et al., 2019; Wang et al., 2019; Araujo et al., 2019; García et al., 2020; Tirloni et al., 2020; Karim et al., 2021; Couto et al., 2021; Oleaga et al., 2021; Pérez-Sánchez et al., 2021). Ribeiro and Mans (2020) pinpointed the large diversity of tick salivary proteins and indicated that “a formidable task of annotation of these transcripts awaits”. These authors also indicated that at least 136 families of tick salivary secreted protein families have been identified, and that they change among species, rendering the annotation a tremendous work. Proteins in sialome of ticks have been used for tracking the molecular evolution of these arthropods (Mans and Neitz, 2004; Mans et al., 2008) or to infer mechanisms of gene evolution, duplication, and function (Mans et al., 2017).

Even if our study is intended only to open the door to a functional approach to the sialome of ticks, we noticed interesting analytical results. The high number of proteins in tick saliva was initially thought to be correlated with their long feeding time, as a response to the hemostatic host response, its tissue repair and the immune response (Ribeiro and Mans, 2020). However, deep sequencing of salivary transcriptomes at different times of feeding ticks uncovered that the tick changes its salivary repertoire frequently, within hours, in a process named “sialome switching” (Valenzuela et al., 2002; Karim and Ribeiro, 2015; Perner et al., 2018b). Ribeiro and Mans (2020) hypothesized that ticks would evade the immune response by switching a number of key proteins, something that we consider a robust hypothesis. The “translation” of that protein switching into biological processes exposed how the corresponding LL processes correlate with the lack of proteins. In example, Rs ticks in G3 lack 430 proteins, and 812 LL processes, but TL processes are not deeply altered. This is of importance because it implies the existence of a balancing mechanism: either new proteins with the same function are incorporated to the cocktail, or the remaining proteins are under- or over-expressed, allowing TL to have similar PageRank values, an extreme not evaluated in this study. Even considering the possible absence or the unreliable selection of proteins and thus LL, the major physiological processes remain because TL are redundant (several pathways of LL drive to the same TL). It thus results that LL may explain how the fine machinery of the tick salivary glands physiology is tuned; the overview at the coarser resolution (the TL) explains the feeding under a wider and rougher perspective. With our acknowledgement of the gaps in this study and the ability of studies on particular gene regulation in tick’s salivary glands, we firmly support the usefulness of our evaluation “in the whole perspective” as long as the annotation of the sialome continue its slow advance.

The phases of tick feeding could be described as: (I) attachment, (II) wound site formation, (III) feeding, (IV) mating, (V) repletion (Sonenshine, 1991). Salivar glands accelerate the synthesis of the proteins after the onset of the feeding. Then after there is a slightly decreases of the production around the first three days, in which the tick barely ingest blood. Later on, the salivary glands secretion increases, this copious discharge of proteins is maintained until the end of the meal, and after the mating a large number of proteins disappear (Sonenshine, 1991). All these morphological and physiological details clearly overlap with our findings about LL, supporting our results. The tight overlap between protein changes and feeding stage has been described de Castro et al. (2017) among others. However, argasids merely feed for around one hour. Also, argasids do not produce cement. Therefore, the differences between Rs and Or were expected but are striking. Argasids ticks are fast feeders. However, blood digestion is slower, even in ovipositing females, with little changes over several weeks (Sonenshine, 1991). The results obtained here are suggestive of (i) a large difference of proteins between argasid and ixodid ticks, (ii) the presence of some TL in the argasid not found in the ixodid, and (iii) the similar biological processes found in the SG and gut of the argasid. The last extreme could not be checked in the ixodid, because the lack of data from gut. The observed results could suggest either that the argasid might have a partial copy of biological processes of the SG in the gut, expressed at different rates, or the result of an unreliable protocol of selection of proteins (referred to in our comments above about probable gaps in the current study). We would like to encourage further research on this topic, in both ixodid and argasid ticks,

The proof-of-concept using the “response to stress” chain of processes showed also interesting analytical results. First, to note that some LL are active at defined periods of time. This supports the strategy by Tirloni et al. (2020) grouping female ticks according to weight and not the time of feeding; these authors managed to obtain homogeneous groups, with some overlapping in its physiology (like G4 and G5). But data also confirms that blood intake in ixodid ticks has three well defined periods as already described (Sonenshine, 1991) corresponding to our findings regarding small differences in the gland physiology in several of the analyzed periods. Second, this result confirms that prominent LL are mainly active at defined phases of feeding. In example, the lectin receptor signaling pathway is highly active at some periods (with medium activity at other phases). The C-type lectin receptors have an important role in orchestrating the induction of signaling pathways that regulate adaptive immune responses (Geijtenbeek and Gringhuis, 2009) and are well expressed at defined periods of engorgement. The complete response to stress pathway is composed by LL responding to heat, to cold, to the repair of DNA breaking, and to viral and bacterial protection. These results have a solid logic behind and indicate that the proposed method managed to obtain critical features in the functional structure of the stress pathway (shown here only as an example). We firmly think that the handling of a complete proteome (other than inclusion of poorly characterized proteins) is unpractical, and that the study of portions of the LL could produce a better selection of the proteins to be included in the network, as a detailed chain of events.

The prevailing approach to the study of the tick sialome is “protein-centric”, focused on the characterization of families of proteins, which is revealing an unexpected number of details (i.e., Wang et al., 2019). We however think that a functional approach is necessary to understand how tick salivary glands work. A network approach seems to be the best method to obtain such a pragmatic view, even if we only partially did in this study. Networks are now prevalent to explain interactions and a solid mathematical background is behind them (Benjamin et al., 2015; Pilosof et al., 2017). PageRank has been chosen as the best index of the dynamics of biological processes: it is an indicator of the activity of the process, since it is reactive not only to the number of proteins involved in the process, but also to its expression. In a plain language, PR expresses how “cool” or “popular” is a process as the result of the proteins expressed. The network construct allows other multiple approaches, most prominently the “attack to the network” and its resilience. It has been demonstrated that some nodes in a complex network are “key stones” for many links to other nodes (Lhomme, 2015). Methods exist to selectively remove these nodes and evaluate the impact on the indexes defining the network.

We acknowledge that the annotation of transcripts in the tick sialome is a formidable task, and that studies of interactions among proteins are providing the “omics” behind the study of sialome with exceptionally interesting data. We claim for a more functional view. Behind the few examples explained in this study, some of them to be confirmed b future experiments, there is a wide field of research that could be based on *de novo* sequenced datasets or existing data. The power and the robustness of these methods would allow an impaired view of the physiology of any tick organ.

## Data Availability Statement

The original contributions presented in the study are included in the article/**Supplementary Material**. Further inquiries can be directed to the corresponding author.

## Author Contributions

NF-R did the literature search, obtained the data, and did the cleaning of the datasets and the queries to public repositories; she did the main statistical calculations, prepared several figures from scripts in R, and wrote parts of the draft document. AE-P prepared the networks, wrote parts of the draft, outlined several figures, and wrote some of the R scripts. All authors contributed to the article and approved the submitted version.

## Conflict of Interest

The authors declare that the research was conducted in the absence of any commercial or financial relationships that could be construed as a potential conflict of interest.

The reviewer LS declared a past co-authorship with one of the authors AE-P to the handling editor.

## Publisher’s Note

All claims expressed in this article are solely those of the authors and do not necessarily represent those of their affiliated organizations, or those of the publisher, the editors and the reviewers. Any product that may be evaluated in this article, or claim that may be made by its manufacturer, is not guaranteed or endorsed by the publisher.

## Supplementary Material

Supplementary Table 1 The list of the LL processes used in this study. Each column of the file represents one group and is labelled at the top. Unfed, G1 to G6, correspond with the feeding groups of *R. sanguineus*. Data for salivary glands or gut of *Ornithodoros rostratus* are labelled as SG or Gut.

Supplementary File 1 The complete networks for the LL of the groups G1 to G6 of *R. sanguineus*. Also included are the proteins and LL processes of *O. rostratus* and the proof-of-concept of the mini-networks derived from the main pathway “Response to stress”. All the data are in Gephi format, which can be downloaded at http://gephi.org.

Supplementary File 2 The complete networks for the TL of the groups G1 to G6 of *R. sanguineus*. Also included are the proteins and TL processes of *O. rostratus*. All the data are in Gephi format, which can be downloaded at http://gephi.org.
