# Supplementary material for: Scenes From Tick Physiology: Proteins of Sialome Talk About Their Biological Processes
Source: Front Cell Infect Microbiol. 2022 Jan 4;11:767845. doi: 10.3389/fcimb.2021.767845 (PMC8765405; doi:10.3389/fcimb.2021.767845)
Supplement: Supplementary File 1 — The complete networks for the LL of the groups G1 to G6 of R. sanguineus. Also included are the proteins and LL processes of O. rostratus and the proof-of-concept of the mini-networks derived from the main pathway “Response to stress”. All the data are in Gephi format, which can be downloaded at http://gephi.org. [file DataSheet_1.pdf]

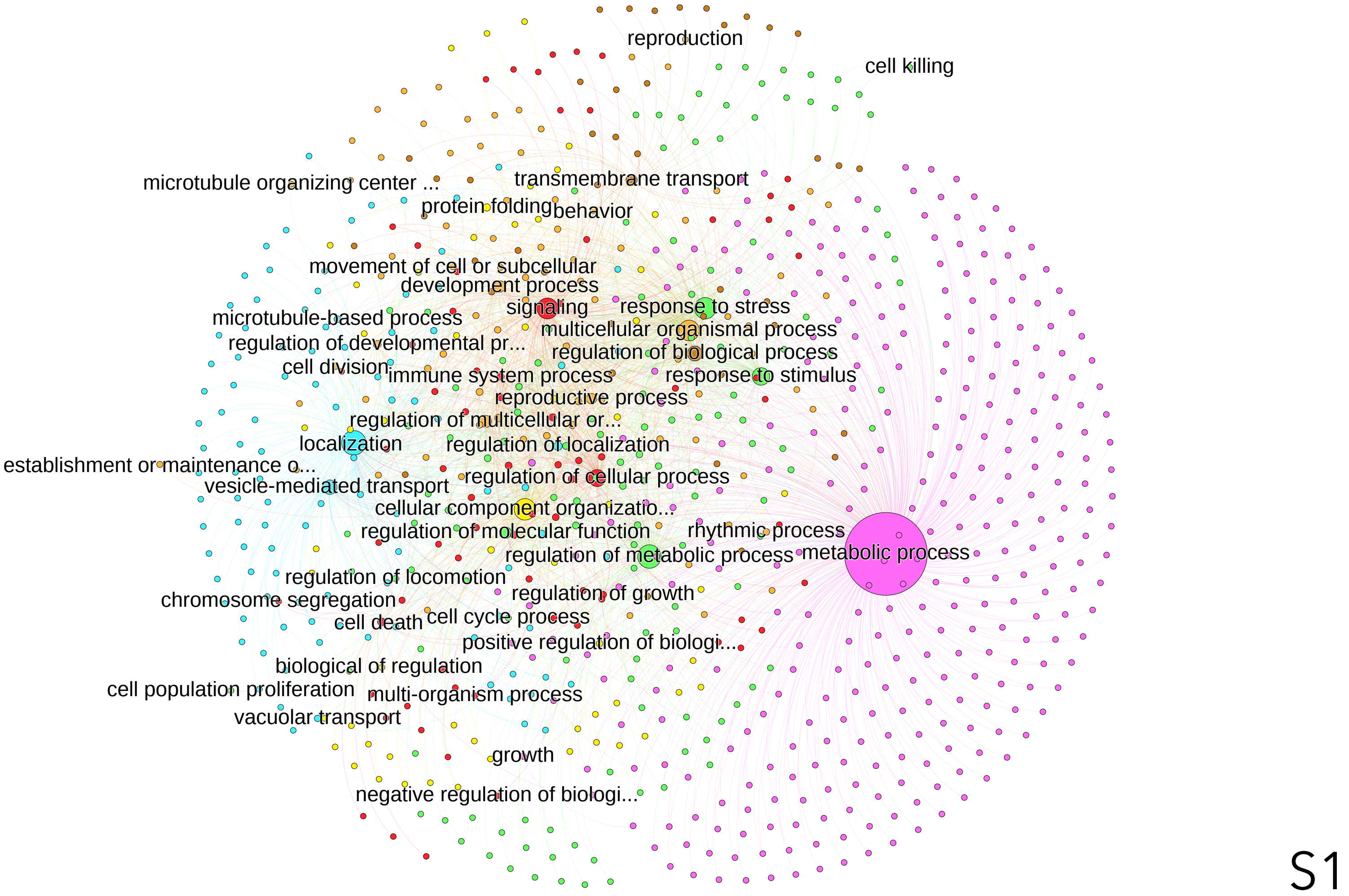

Supplementary Figure 1. The network of unfed *Riphicephalus sanguineu*s s.l. Each node represents either a protein or a top-level process. Only processes are labeled. The colours express the modularity (groups of proteins and processes that interact more frequently than with the rest). The size of each node is proportional to PageRank. The width of the line is proportional to strength of the link

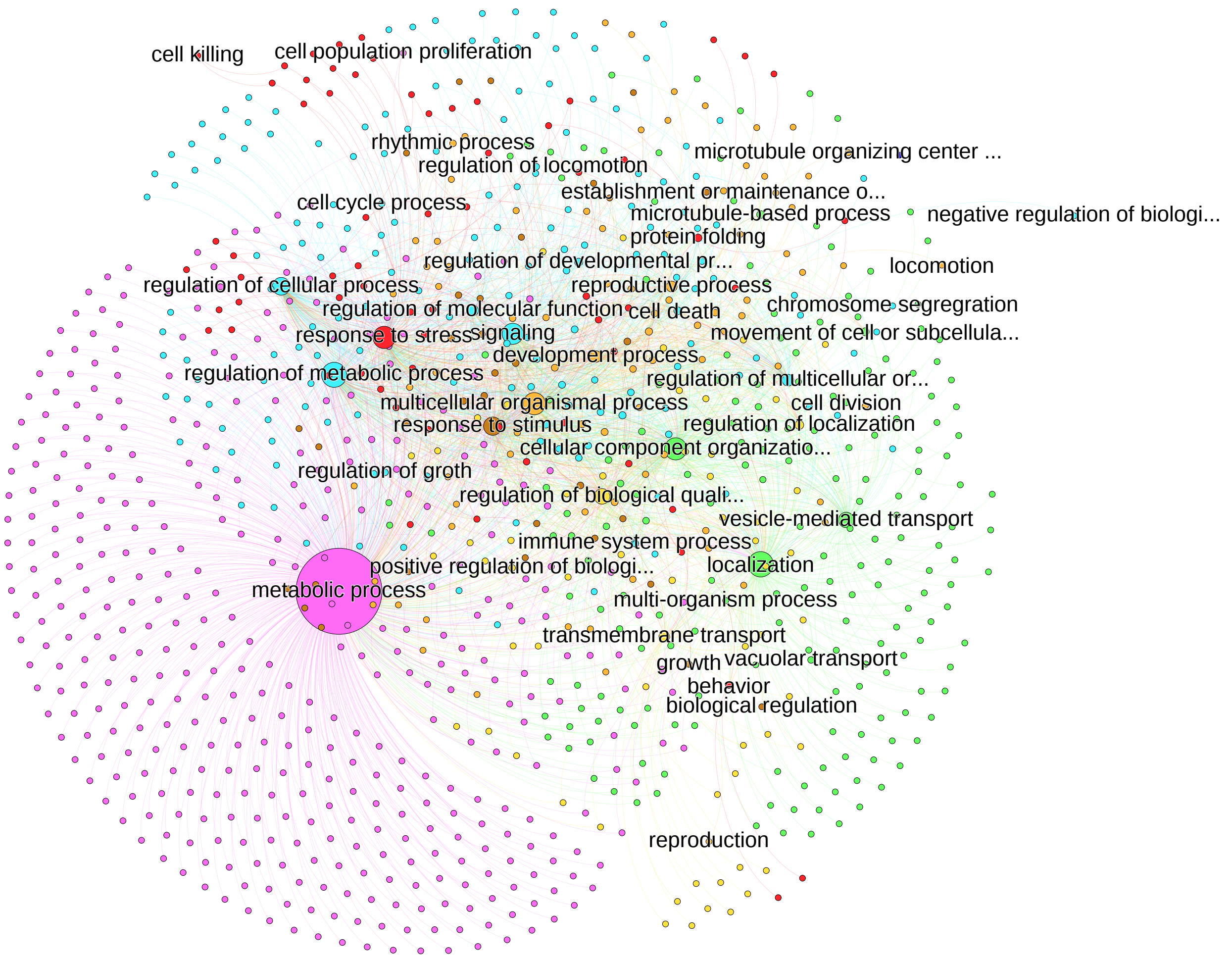

S2

Supplementary Figure 2. The network of feeding *Riphicephalus sanguineus* s.l. tick with average weight 1.8 mg collected at day 2 of feeding. Each node represents either a protein or a top level process. Only processes are labeled. The colours express the modularity (groups of proteins and processes that interact more frequently than with the rest). The size of each node is proportional to PageRank. The width of the line is proportional to strength of the link.

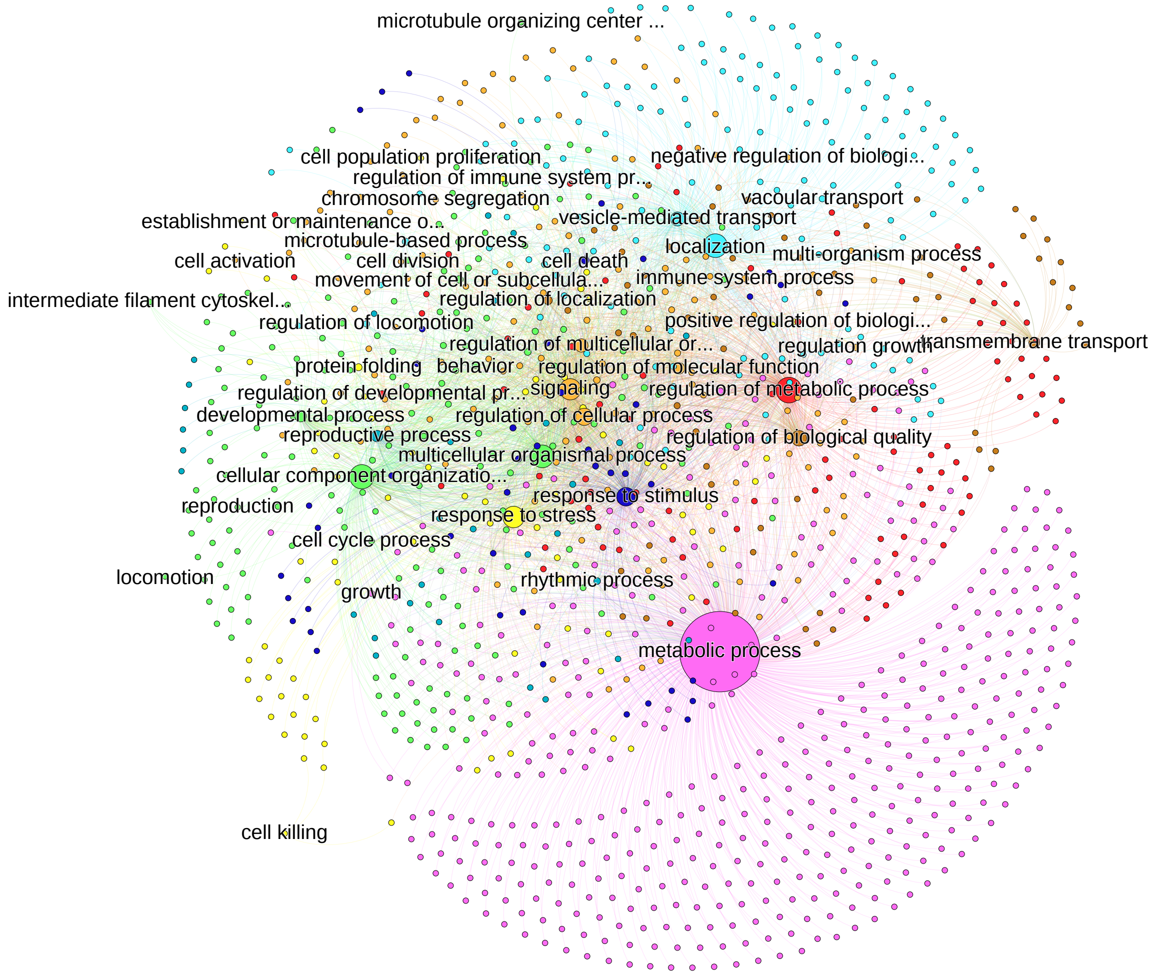

S3

Supplementary Figure 3. The network of feeding *Riphicephalus sanguineus* s.l. tick with average weight 3.6 mg collected at day 6 of feeding. Each node represents either a protein or a top level process. Only processes are labeled. The colours express the modularity (groups of proteins and processes that interact more frequently than with the rest). The size of each node is proportional to PageRank. The width of the line is proportional to strength of the link.

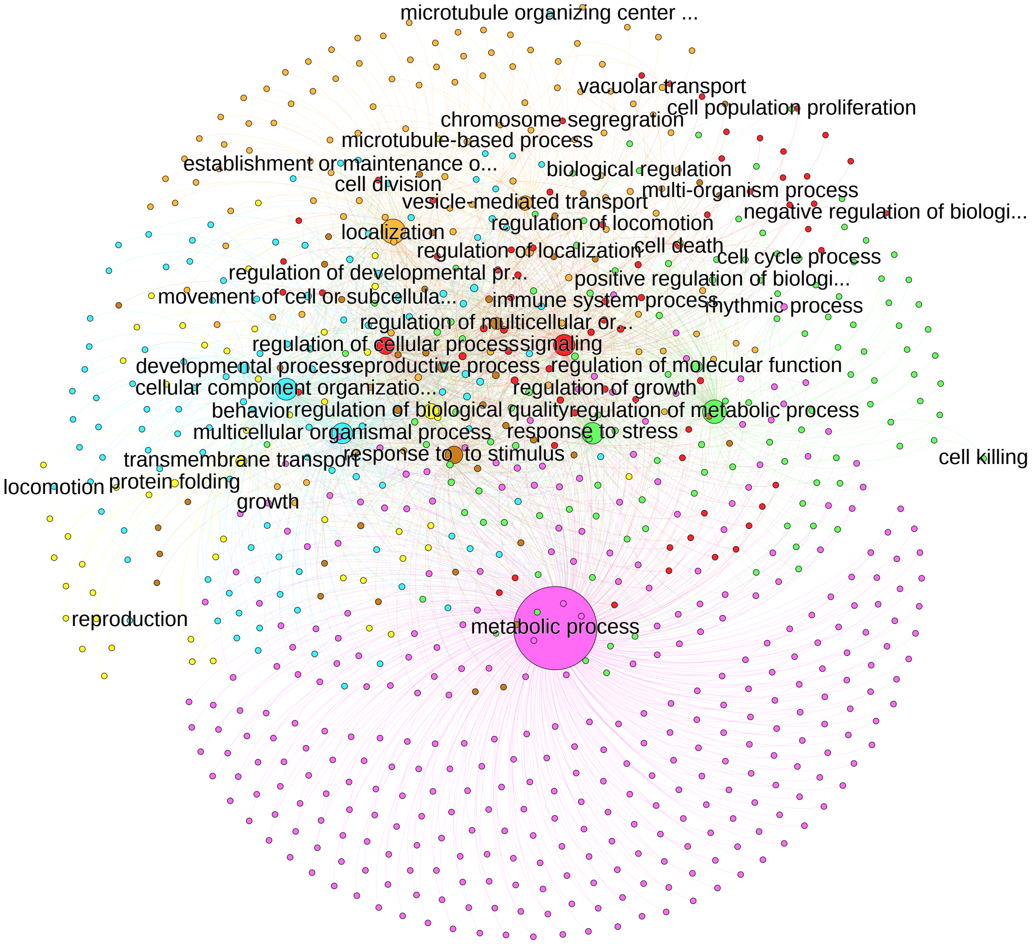

S4

Supplementary Figure 4. The network of feeding *Riphicephalus sanguineu*s s.l. tick with average weight 7 mg collected at day 6 of feeding. Each node represents either a protein or a top level process. Only processes are labeled. The colours express the modularity (groups of proteins and processes that interact more frequently than with the rest). The size of each node is proportional to PageRank. The width of the line is proportional to strength of the link.

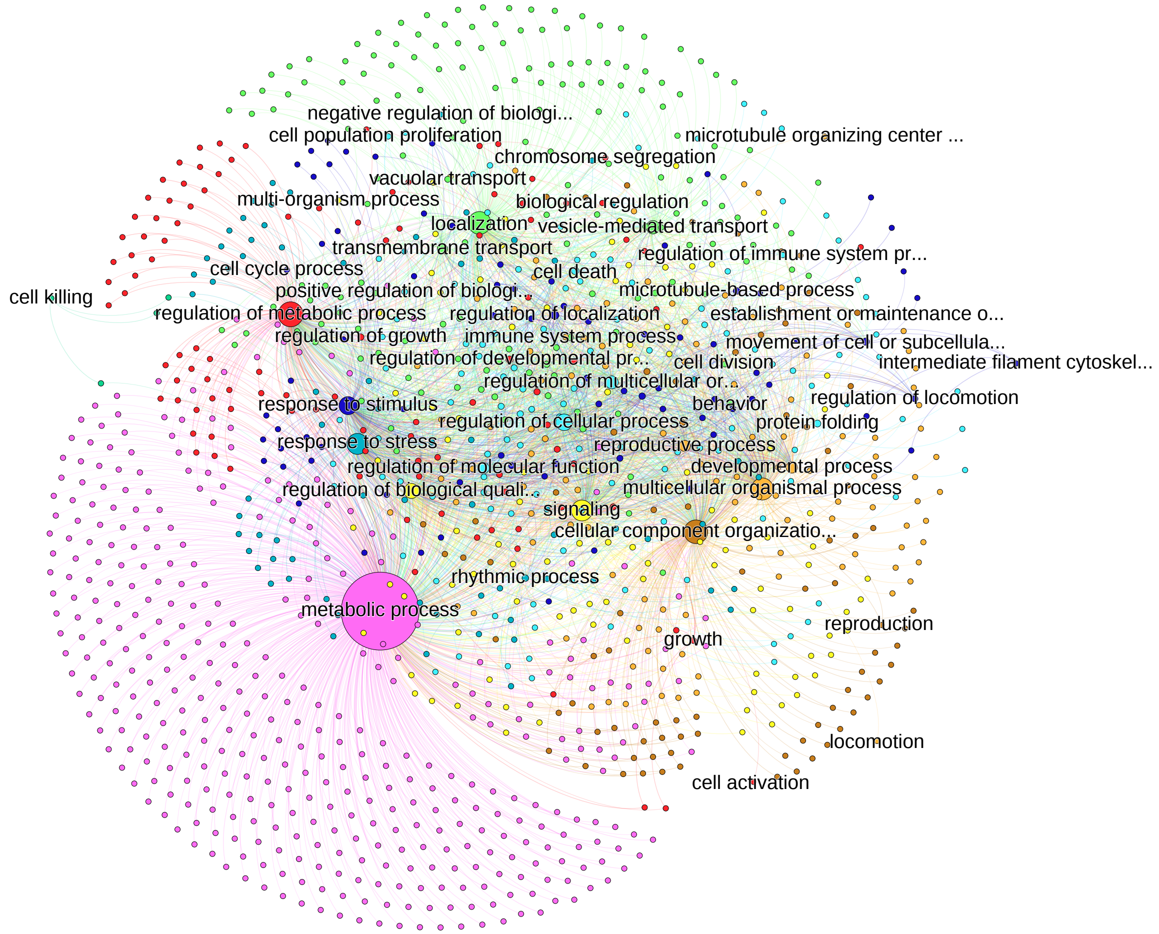

S5

Supplementary Figure 5. The network of feeding *Riphicephalus sanguineu*s s.l. tick with average weight 10.9 mg collected at day 8 of feeding. Each node represents either a protein or a top level process. Only processes are labeled. The colours express the modularity (groups of proteins and processes that interact more frequently than with the rest). The size of each node is proportional to PageRank. The width of the line is proportional to strength of the link.

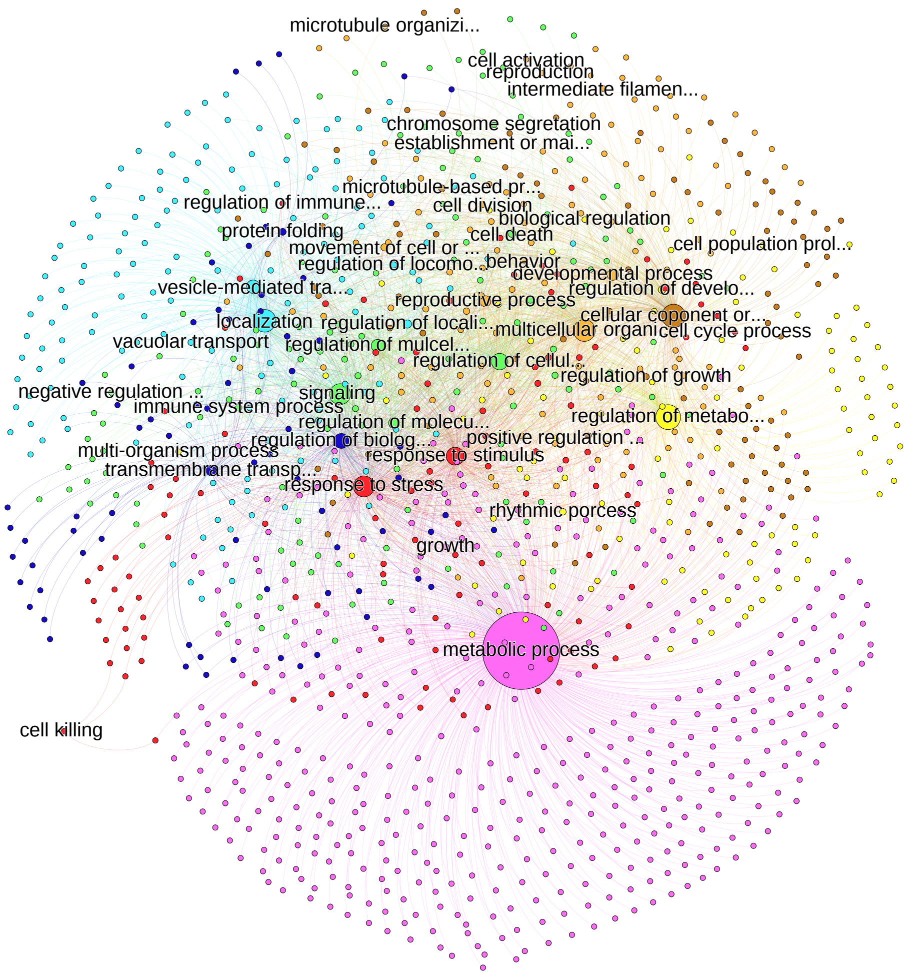

Supplementary Figure 6. The network of feeding *Riphicephalus sanguineus* s.l. tick with average weight 24 mg collected at day 8-11 of feeding. Each node represents either a protein or a top level process. Only processes are labeled. The colours express the modularity (groups of proteins and processes that interact more frequently than with the rest). The size of each node is proportional to PageRank. The width of the line is proportional to strength of the link.

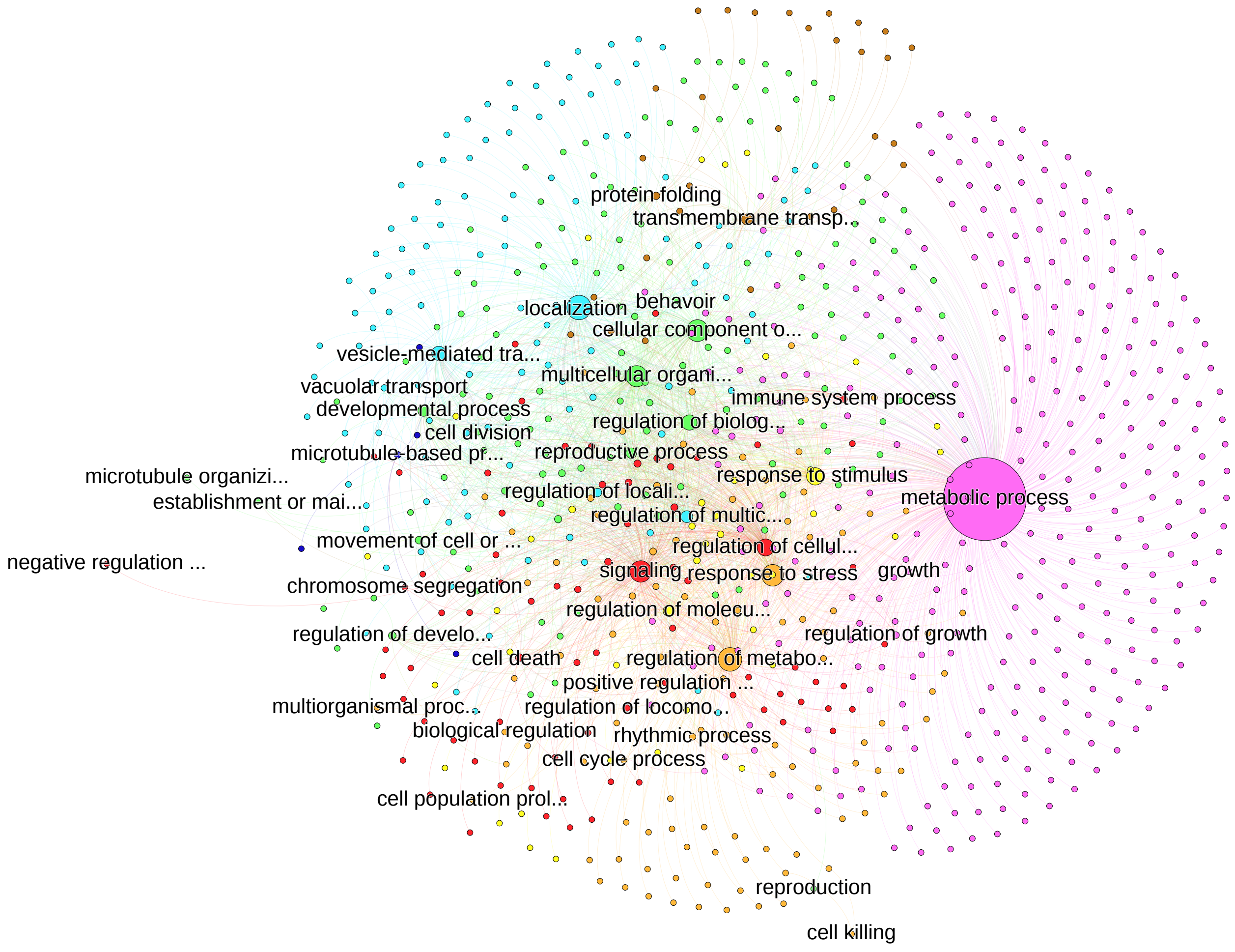

S7

Supplementary Figure 7. The network of feeding *Riphicephalus sanguineu*s s.l. tick with average weight 36 mg collected at day 6, 10, 13 of feeding. Each node represents either a protein or a top level process. Only processes are labeled. The colours express the modularity (groups of proteins and processes that interact more frequently than with the rest). The size of each node is proportional to PageRank. The width of the line is proportional to strength of the link.

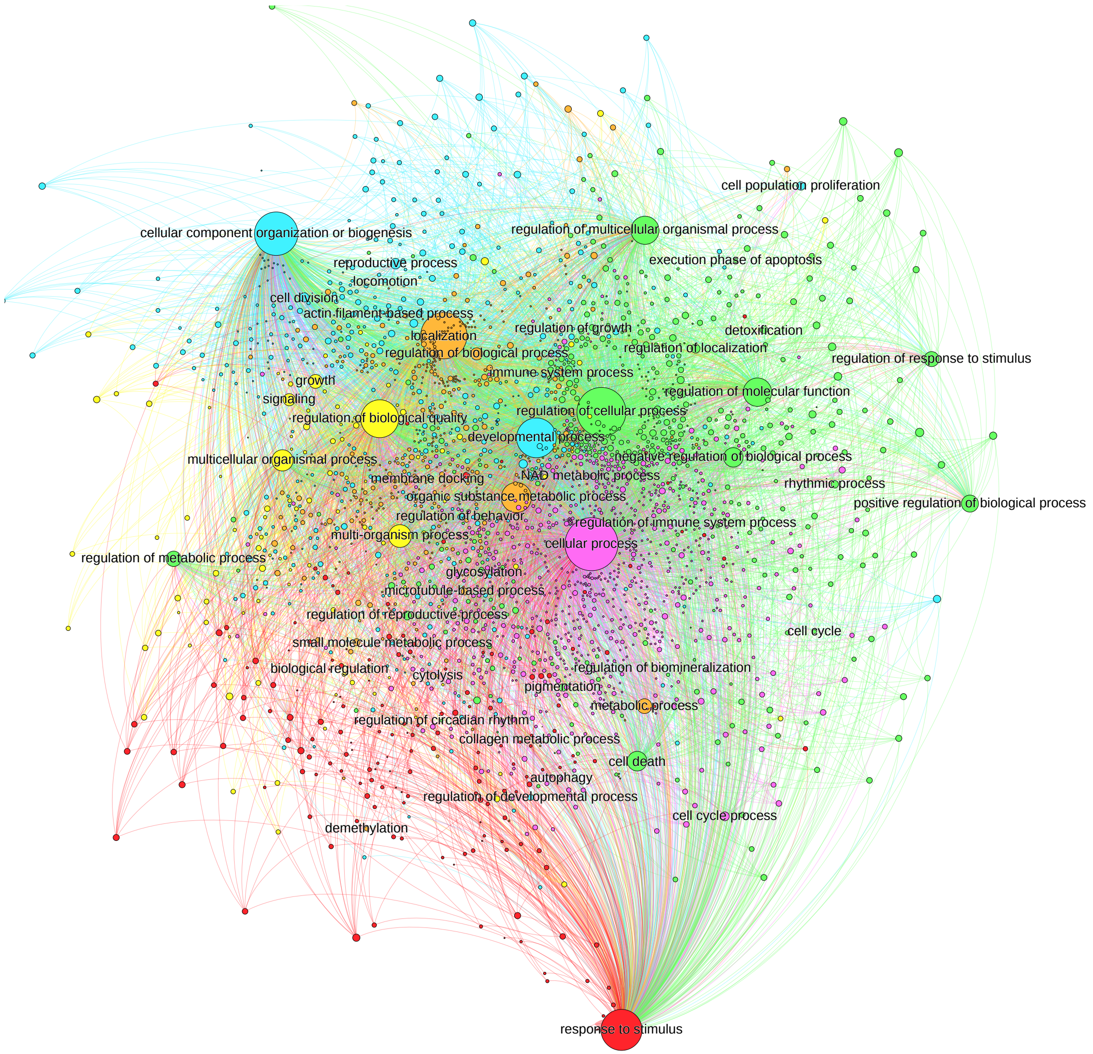

S8

Supplementary Figure 8. The network of salivary gland of feeding *Ornithodoros rostratus* tick. Each node represents either a protein or a top level process. Only processes are labeled. The colours express the modularity (groups of proteins and processes that interact more frequently than with the rest). The size of each node is proportional to PageRank. The width of the line is proportional to strength of the link.

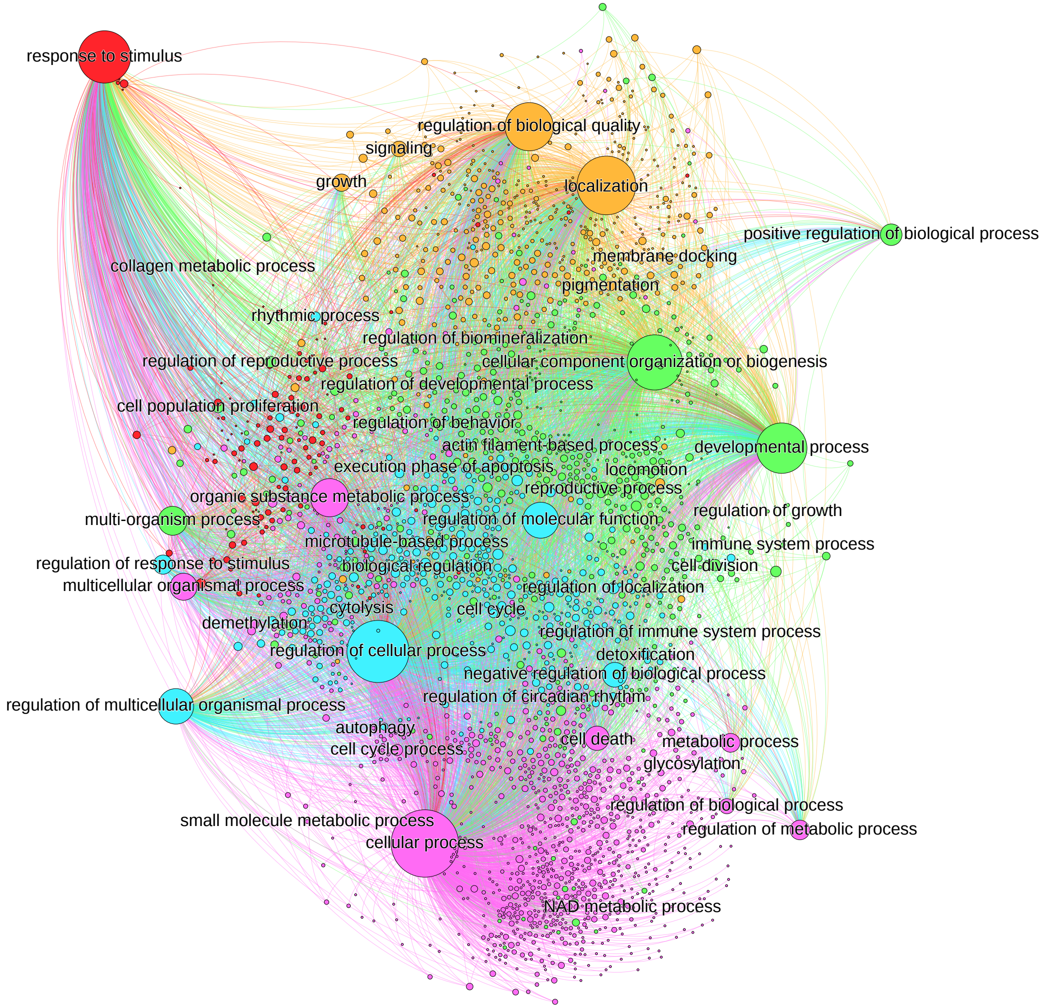

S9

Supplementary Figure 9. The network of gut of feeding *Ornithodoros rostratus* tick. Each node represents either a protein or a top level process. Only processes are labeled. The colours express the modularity (groups of proteins and processes that interact more frequently than with the rest). The size of each node is proportional to PageRank. The width of the line is proportional to strength of the link.
